# Comparison of range-of-motion and variability in upper body movements between transradial prosthesis users and able-bodied controls when executing goal-oriented tasks

**DOI:** 10.1186/1743-0003-11-132

**Published:** 2014-09-06

**Authors:** Matthew J Major, Rebecca L Stine, Craig W Heckathorne, Stefania Fatone, Steven A Gard

**Affiliations:** Northwestern University Prosthetics-Orthotics Center, Northwestern University Feinberg School of Medicine, 680, Lake Shore Dr, Chicago, 60611 IL USA; Jesse Brown VA Medical Center, 820 S, Damen Ave, Chicago, 60612 IL USA

**Keywords:** Upper limb, Transradial amputation, Kinematics, Trunk, Prosthesis

## Abstract

**Background:**

Current upper limb prostheses do not replace the active degrees-of-freedom distal to the elbow inherent to intact physiology. Limited evidence suggests that transradial prosthesis users demonstrate shoulder and trunk movements to compensate for these missing volitional degrees-of-freedom. The purpose of this study was to enhance understanding of the effects of prosthesis use on motor performance by comparing the movement quality of upper body kinematics between transradial prosthesis users and able-bodied controls when executing goal-oriented tasks that reflect activities of daily living.

**Methods:**

Upper body kinematics were collected on six able-bodied controls and seven myoelectric transradial prosthesis users during execution of goal-oriented tasks. Range-of-motion, absolute kinematic variability (standard deviation), and kinematic repeatability (adjusted coefficient-of-multiple-determination) were quantified for trunk motion in three planes, shoulder flexion/extension, shoulder ab/adduction, and elbow flexion/extension across five trials per task. Linear mixed models analysis assessed between-group differences and correlation analysis evaluated association between prosthesis experience and kinematic repeatability.

**Results:**

Across tasks, prosthesis users demonstrated increased trunk motion in all three planes and shoulder abduction compared to controls (p ≤ 0.004). Absolute kinematic variability was greater for prosthesis users for all degrees-of-freedom irrespective of task, but was significant only for degrees-of-freedom that demonstrated increased range-of-motion (p ≤ 0.003). For degrees-of-freedom that did not display increased absolute variability for prosthesis users, able-bodied kinematics were characterized by significantly greater repeatability (p ≤ 0.015). Prosthesis experience had a strong positive relationship with average kinematic repeatability (r = 0.790, p = 0.034).

**Conclusions:**

The use of shoulder and trunk movements by prosthesis users as compensatory motions to execute goal-oriented tasks demonstrates the flexibility and adaptability of the motor system. Increased variability in movement suggests that prosthesis users do not converge on a defined motor strategy to the same degree as able-bodied individuals. Kinematic repeatability may increase with prosthesis experience, or encourage continued device use, and future work is warranted to explore these relationships. As compensatory dynamics may be necessary to improve functionality of transradial prostheses, users may benefit from dedicated training that encourages optimization of these dynamics to facilitate execution of daily living activity, and fosters adaptable but reliable motor strategies.

**Electronic supplementary material:**

The online version of this article (doi:10.1186/1743-0003-11-132) contains supplementary material, which is available to authorized users.

## Background

The musculoskeletal architecture of the upper body (i.e., trunk and arms) contains redundant degrees-of-freedom (DoFs) from which the central nervous system (CNS) has the benefit of selecting various task-equivalent motor strategies and associated inter-joint coordination patterns to satisfy task requirements [1–3]. The advantage of this redundancy is that the neuromuscular system is highly adaptable and capable of accounting for a loss in DoFs due to pathology [4–9]. This motor adaptability is of critical advantage for individuals with transradial limb loss in the context of task performance, as typical upper limb prostheses do not replace the active DoFs distal to the elbow inherent to intact physiology, including forearm pronation/supination and wrist flexion/extension [10]. There is substantial evidence to suggest that due to loss of active DoFs, users of transradial prostheses employ compensatory movements predominantly at the trunk and shoulder to execute goal-oriented tasks [5, 6]. Studies have further emphasized the impact of forearm and wrist movement on compensatory motions during execution of goal-oriented tasks by demonstrating that able-bodied individuals exhibit similar compensatory motions as transradial prosthesis users (e.g., lateral trunk flexion) when forearm and wrist movement is restricted [11, 12], and fixing the prosthetic wrist in different flexion angles affects prosthetic arm compensatory movements (e.g., shoulder anteversion and abduction) [13].

Despite indication that compensatory motions may result in overuse injuries [12, 14], the opinion exists that this behavior should not be discouraged when operating an upper limb prosthesis as it demonstrates adaptability given the functional limitations of the device, and appropriate strategies should be identified to facilitate task execution [15]. The key to control when using a transradial prosthesis is to properly manage the remaining DoFs [5], and studies indicate that the CNSs of prosthesis users create accurate internal models of the affected arm through residual sensory feedback and utilize adapted motor control strategies (i.e., coordinated joint torques and geometry) for task execution [16–19]. Importantly, evidence suggests that although upper limb prosthesis users adapt to novel task environments, they demonstrate increased motor variability, but dedicated experience and training with the device may facilitate long-term motor adaptation and increased kinematic repeatability [18, 20, 21]. Therefore, the ultimate goal of rehabilitation therapy to enhance motor learning and refine these movements through practice via task repetition is reasonable [18–20, 22, 23]. A certain level and type of kinematic variability may be considered an asset to upper limb prosthesis users for the purpose of exploiting motor flexibility that may aid task execution [24], but the amount of variability associated with upper body movement in this group compared to able-bodied controls has not been adequately explored. Furthermore, dependable motor behavior, as reflected by reduced movement variability that results from appropriate prosthetic training, may reflect better prosthetic control, which could prevent user dissatisfaction and device abandonment [25–27]. However, despite the common therapeutic goal of enhancing movement quality, there still remains a dearth of information on the characteristics of upper body compensatory movements and associated variability of transradial prosthesis users when executing goal-oriented tasks, and the relationship of these dynamics with device experience [20]. This is partially due to the paucity of upper limb prosthetic research studies incorporating methods for the characterization of upper body kinematics as a form of outcome measurement to assess performance [15, 20].

The objectives of this study were to compare upper body movements and their associated variability between able-bodied individuals and transradial prosthesis users during execution of goal-oriented tasks that reflect activities of daily living (ADLs), and to assess relationships between movement variability and prosthesis experience. We hypothesize that: 1) prosthesis users will display altered range-of-motion (RoM) at the trunk and shoulder compared to able-bodied individuals to compensate for a loss in active distal DoFs, 2) prosthesis users will display increased within-subject movement variability as they recruit compensatory motions to execute novel goal-oriented tasks, and 3) movement repeatability of prosthesis users has a positive direct relationship with prosthesis experience. Importantly, upper limb prosthesis use performance has traditionally been assessed through the use of clinical outcome measures [28–30]. Although these measures provide useful clinically-relevant information, they only provide information on functional outcome and are unable to assess the contribution of movement quality to overall performance [23]. The results from this study will allow for improved insight into the movement quality associated with upper limb prosthesis user performance, contribute to quantification of compensatory movements when executing goal-oriented tasks, and enhance understanding of how the motor strategies of prosthesis users differ from able-bodied individuals and are related to device experience.

## Methods

### Participants

Participant inclusion criteria required that they be free of neurological and musculoskeletal pathology (apart from upper limb loss) that would impair upper limb motor control during goal-oriented task execution in a seated position. Participants with limb loss were required to have unilateral loss at the transradial level and currently operate a prosthesis with myoelectric control that would be used during the experiments. A requirement was that the prosthesis be self-suspending with a supracondylar socket design. Ethical approval was obtained from the University Institutional Review Board, and informed consent was provided by each participant prior to data collection.

Six able-bodied individuals (3 male, 3 female, 35 ± 11 years) and seven individuals with limb loss at the transradial level (5 male, 2 female, 49 ± 18 years) participated in this study. Limb loss etiology included traumatic (n = 4) and congenital (n = 3). User devices included the System Electric Hand (n = 1; Otto Bock, Duderstadt, Germany), MyoHand VariPlus Speed Hand (n = 1; Otto Bock), Transcarpal Hand (n = 1; Otto Bock), Motion Control Hand (n = 1; Motion Control, Salt Lake City, UT), i-Limb Ultra Revolution (n = 1; Touch Bionics, Livingston, UK), i-Limb Ultra (n = 1; Touch Bionics), and i-Limb Hand (n = 1; Touch Bionics). For all participants with traumatic amputation, the amputated limb was considered their dominant limb prior to the amputation and the average time since amputation was 9.5 ± 11.0 years. Across all subjects, the average reported prosthesis experience was 20.3 ± 18.1 years. Prosthetic socket comfort was quantified with the socket comfort score [31], which asks users to rate their prosthesis on an ordinal scale from 0 (most uncomfortable socket imaginable) to 10 (most comfortable socket imaginable), and the average score was 8.9 ± 1.1, suggesting a high level of comfort across participants.

### Experimental protocol

Participants were requested to execute five goal-oriented tasks while seated at a table of standard height (73.7 and 28 cm from floor and seat top, respectively). The tasks and associated protocol were adopted from the Southampton Hand Assessment Procedure (SHAP) [32], as this assessment provided a standardized protocol for controlled, repeated performance of goal-oriented tasks that reflect ADLs:

1. Food cutting – picking up and positioning a knife to slice through a small plasticine roll located at midline of the body.
2. Page turning – grasping a page, located on one side of the body, with the hand on the contralateral side and turning the page over to the ipsilateral side as if turning the page of a book.
3. Carton pouring – lifting a carton, located at midline of the body, and emptying the liquid contents into a jar on the contralateral side with minimal spilling.
4. Lifting and transferring a weighted object – lifting a liquid-filled jar, located on one side of the body, with the hand on the contralateral side and transferring to the ipsilateral side over a low-level barrier at midline of the body.
5. Lifting and transferring a tray – lifting a tray, located on the contralateral side of the body, with both hands and transferring to the ipsilateral side over a low-level barrier at midline of the body.

**Figure 1:**
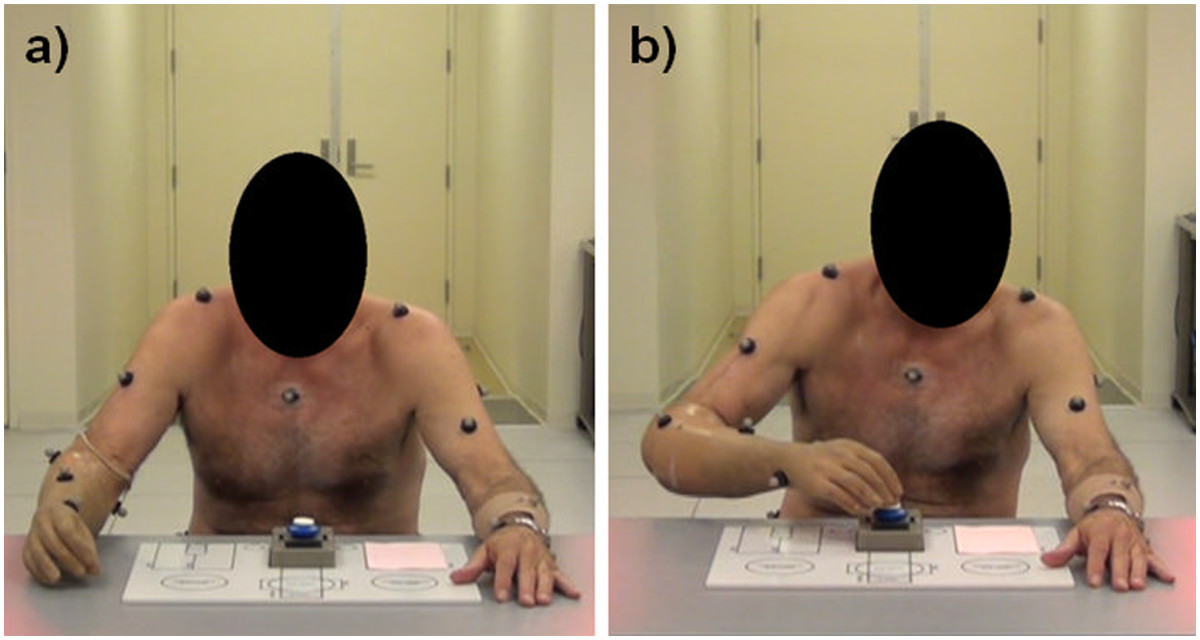
**Image of a prosthesis user in the position prior to the start of task (a), and at the start and end of task execution (b).**

Per the SHAP protocol, participants were requested to complete each task as fast as possible. Tasks did not require participants to position their joints/segments in any particular orientation in space, but only to complete the task requirements. Able-bodied controls and prosthesis users performed these tasks using their non-dominant and prosthetic limb, respectively, for unimanual tasks (page turning, carton pouring, lifting and transferring a weighted object). For the tasks of page turning and lifting and transferring a weighted object and tray, the object was first positioned on the side of the body opposite to the non-dominant or prosthetic limb. Prior to data collection and the start of each task, the participants rested both hands on the table in the same position (Figure 1a). Once the participants were requested to start the task, they struck a large button at midline of the body with their non-dominant or prosthetic limb (Figure 1b) and this event denoted the start of the task. Following execution of each task, the participant again struck the button and this denoted the end of the task. Five trials of each task were performed in random order to minimize acute learning effects [22], as the intent was to observe performance of the desired tasks and not capture changes due to learning through repetition [23]. Participants were permitted practice to familiarize themselves with the task requirements prior to data collection.

### Data collection and analysis

Kinematic data were collected with a digital motion capture system (Motion Analysis Corporation, Santa Rosa, CA) at 120 Hz using a custom upper extremity retro-reflective marker set to define three rigid-body segments (forearm, upper arm, and trunk) and three additional markers on the table surface to define the work plane. A subset of the anatomical locations of retro-reflective markers used in this analysis include: C7 spinous process, sacrum, top of the sternum, bilateral acromion processes, medial and lateral humeral epicondyles, and radial and ulnar styloids. For prosthesis users, the locations of the epicondyle and styloid markers mimicked the relative position of these bony landmarks on the intact arm. Virtual elbow and wrist joint centers were created as the midpoint between the epicondyle and styloid markers, respectively. The forearm was defined as the segment linking the virtual wrist and elbow centers, and the upper arm was defined as the segment linking the virtual elbow center and acromion process marker.

Upper body kinematics (i.e., shoulder flexion/extension, shoulder ab/adduction, elbow flexion/extension, trunk lateral flexion, trunk transverse rotation, and trunk forward flexion) were estimated from the five trials of each task using custom software (Matlab, Natick, MA). A trunk-based local reference frame was calculated using the C7, sacrum, and sternum markers to define the anatomical sagittal, frontal, and transverse planes. Shoulder flexion/extension was defined as the angle between the upper arm and trunk anterior-posterior axis (zero angular displacement defined as perpendicular alignment of the upper arm and trunk axis). Shoulder abduction was defined as the angle between the upper arm and shoulder axis created by the acromion markers (zero angular displacement defined as perpendicular alignment of the upper arm and shoulder axis). Elbow flexion/extension was defined as the angle between the upper arm and forearm (180° of angular displacement was defined as full elbow extension). Trunk lateral flexion was defined as the angle between the trunk inferior-superior axis and table medial-lateral axis (i.e., table width) projected onto the frontal plane (zero angular displacement defined as perpendicular alignment of the trunk and table axes). Trunk transverse rotation was defined as the angle between the shoulder axis and table medial-lateral axis projected onto the transverse plane (zero angular displacement defined as parallel alignment of the shoulder and table axes). Trunk forward flexion was defined as the angle between the trunk inferior-superior axis and table anterior-posterior axis projected onto the sagittal plane (zero angular displacement defined as perpendicular alignment of the trunk and table axes). Kinematic data were filtered with a bidirectional 4th order low-pass Butterworth filter at 6 Hz. DoF angle trajectories were normalized to task completion time (i.e., from the first to second button strike).

For each task, average RoM, absolute kinematic variability and kinematic repeatability of the observed DoF angles across five trials were calculated for each participant. The RoM of each DoF was estimated by subtracting the maximum and minimum recorded angles. Absolute kinematic variability across the five trials was estimated as the average standard deviation (SD) across all time points of the normalized DoF angle trajectory. Kinematic repeatability was estimated by the adjusted coefficient of multiple determination (CMD), also commonly reported as the *R*^*2*^ value [33]. The CMD provides a statistical estimate of the similarity between waveforms and typically ranges between 0 and 1 [33], with values closer to 1 indicating increased repeatability. Although a useful method for assessing kinematic repeatability, limitations with the CMD method have been reported, such as a direct relationship with DoF RoM and lack of information on absolute measurement variability [34, 35]. Consequently, SD is also reported here to complement CMD as a measure of absolute variability and between-group comparisons of CMD are limited to an analysis for each respective DoF [34, 35]. In light of its utility, CMD has successfully been applied to upper body kinematic studies on children with cerebral palsy executing goal-oriented tasks [36–41].

### Statistical analysis

Statistical analyses were conducted using SPSS (IBM, Armonk, NY) and the critical alpha was set at 0.05. Normality of the data was confirmed using the Shapiro-Wilk test. A linear mixed model (LMM) analysis (fixed factor: group, task, group × task; random factor: subject) was used to assess if group classification resulted in individual DoF RoM, SD, and CMD differences. Group means and 95% confidence intervals were calculated for each task to illustrate task-specific differences and group × task interaction effects. An overall CMD value was calculated for each participant by averaging CMD values across DoFs and tasks (as a measure of overall within-subject kinematic repeatability) and a Pearson correlation coefficient was estimated between prosthesis users’ overall CMD values and prosthesis experience.

## Results

**Figure 2:**
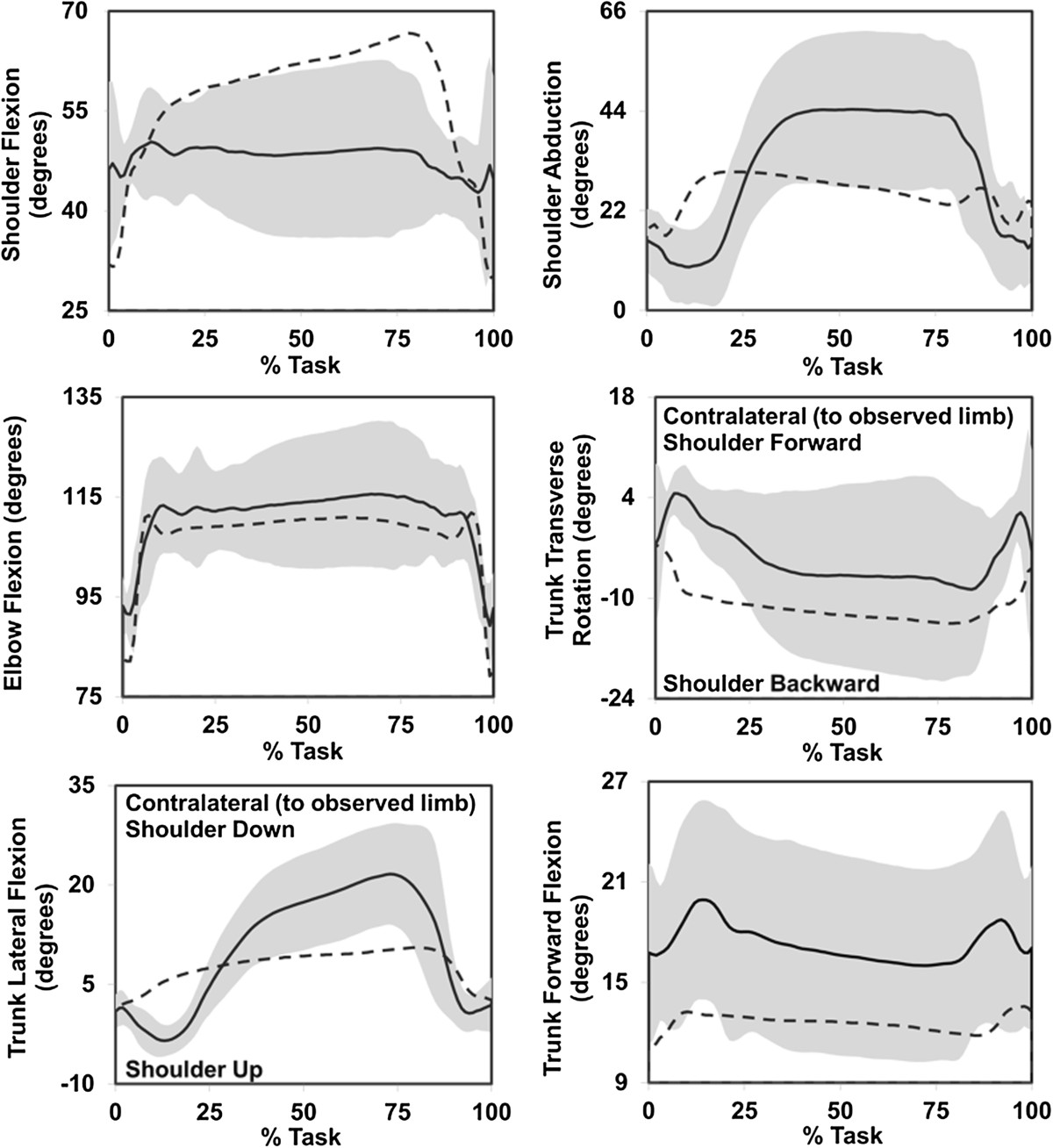
**Group ensemble average kinematic profiles of able-bodied (average = dashed line) and prosthesis users (average = solid line; standard deviation = shaded band) executing task 3 (carton pouring).** Neutral for all DoF angles is 0°, apart from elbow flexion where full extension is 180° and smaller values denote elbow flexion.

**Figure 3:**
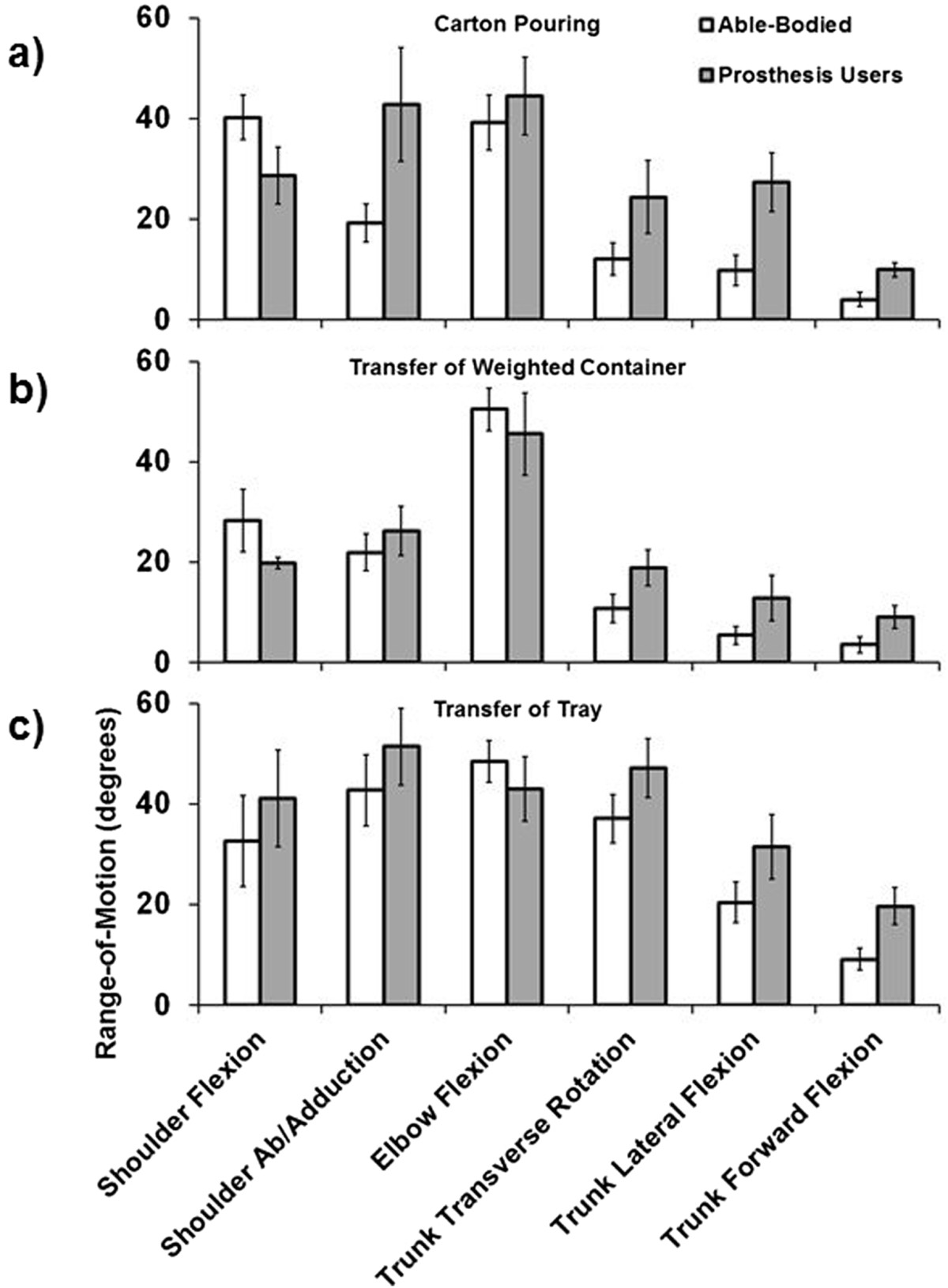
**Group average RoM (maximum angle – minimum angle) for the carton pouring (a), weighted container transfer (b), and tray transfer tasks (c).** Error bars represent the 95% confidence interval.

**Figure 4:**
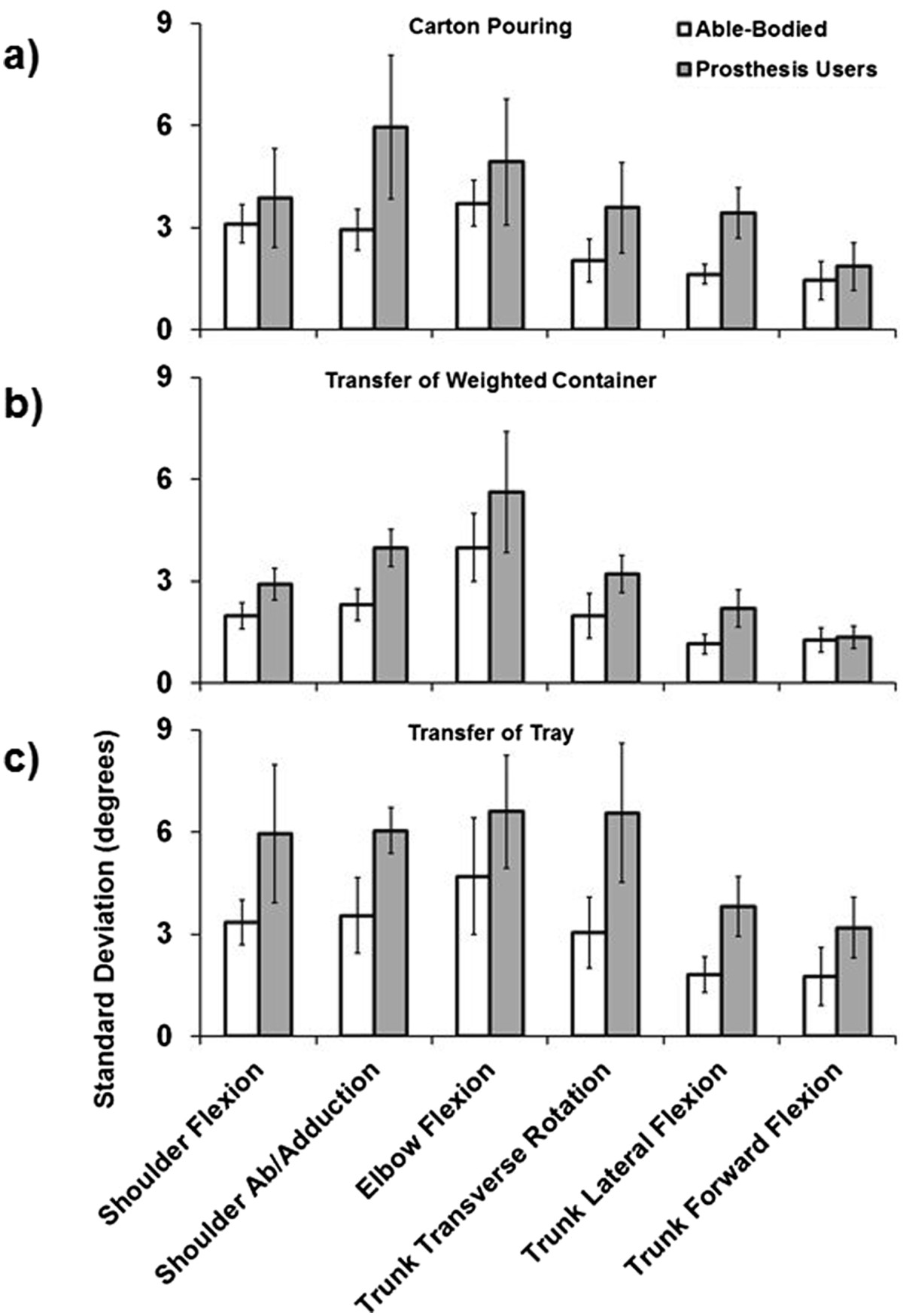
**Group average SD for the carton pouring (a), weighted container transfer (b), and tray transfer tasks (c).** Error bars represent the 95% confidence interval.

**Figure 5:**
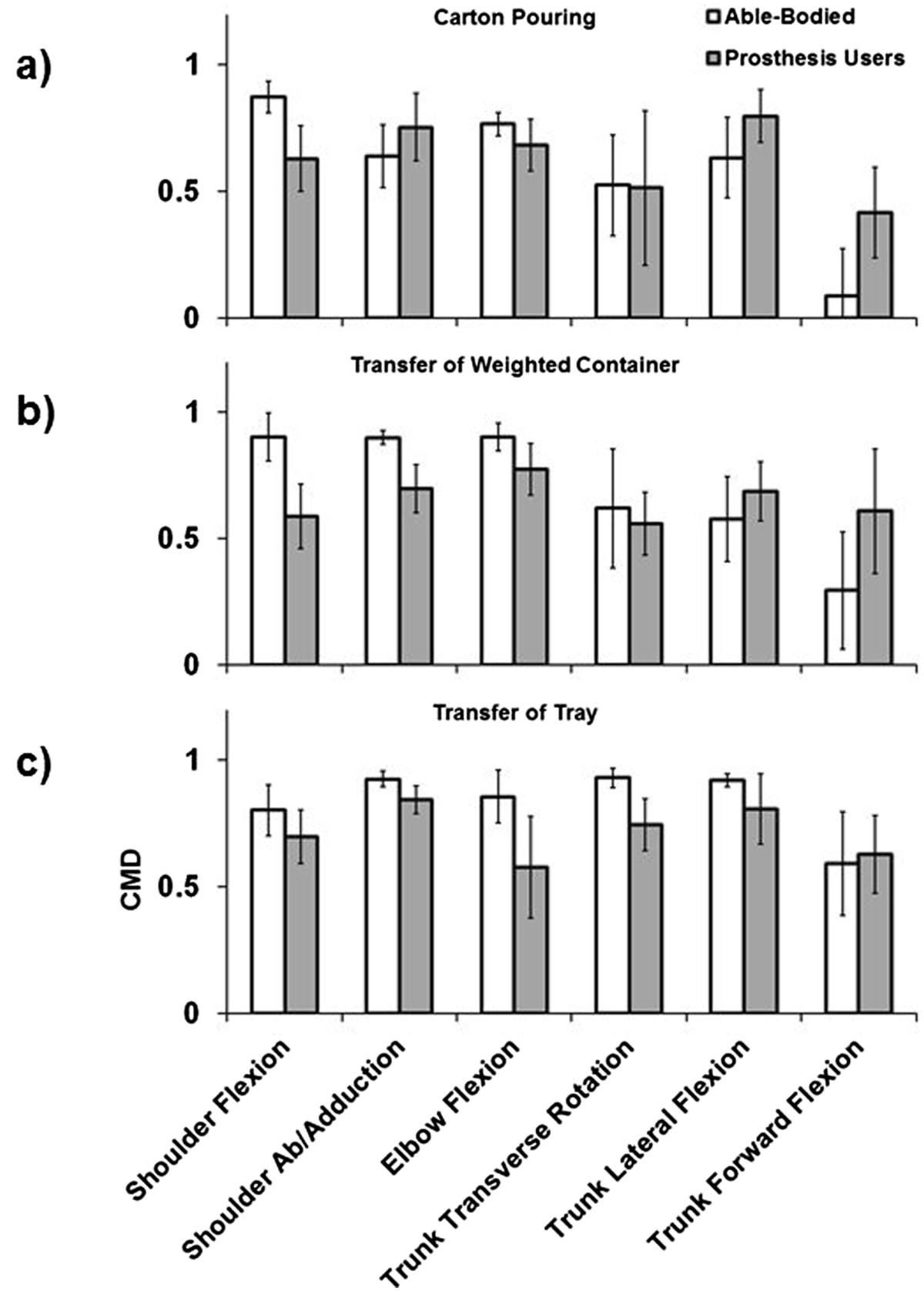
**Group average CMD for the carton pouring (a), weighted container transfer (b), and tray transfer tasks (c).** Error bars represent the 95% confidence interval.

The majority of prosthesis users were unable to routinely execute tasks 1 (food cutting) and 2 (page turning) as required by the protocol due to their inability to maintain grip of the knife and fully rotate the page from front to back, respectively, with their prostheses. Consequently, data from these tasks were eliminated from the analysis. Additionally, one prosthesis user was unable to execute task 5 (tray lift and transfer) due to inadvertent opening of the prosthetic hand and these data are missing from the analysis. To aid in visualization of kinematic results, an example of group ensemble average kinematics of task 3 (carton pouring) is presented in Figure 2. Group data for RoM, SD, and CMD of tasks 3, 4, and 5 are displayed in Figures 3, 4, and 5, respectively, and the LMM statistical results are displayed in Table 1.

Results indicate that prosthesis users demonstrate a significant increase in shoulder abduction, trunk transverse rotation, trunk lateral flexion, and trunk forward flexion RoM. Additionally, for prosthesis users, all of the DoFs with increased RoM apart from trunk forward flexion also displayed increased absolute kinematic variability as reflected by increased average SD. For shoulder flexion/extension, elbow flexion/extension, and trunk forward flexion, which did not display differences in absolute kinematic variability (SD), able-bodied controls demonstrated increased kinematic repeatability as reflected by greater CMD values. Few interaction effects (group × task) were revealed by the statistical analyses, and these were primarily limited to RoM of shoulder flexion/extension and abduction, and trunk lateral and forward flexion (see Figures 3, 4, and 5), demonstrating that these DoFs RoM varied between groups based on task. Overall CMD values of the prosthesis users were strongly correlated with prosthesis experience in years (r = 0.790, p = 0.034) with an average of 0.67 ± 0.07 (range: 0.54 to 0.74), while the average able-bodied CMD value was 0.71 ± 0.03 (range: 0.67 to 0.74).

**Table 1:** **Linear mixed model results for group and group × task interaction effects for each DoF**

		Group effect		Group × Task interaction effect	
Degrees-of-freedom	Parameter	F-value	p-value	F-value	p-value
**Shoulder flexion/extension**	RoM	1.468	0.251	6.613*	0.006*
	SD	4.739	0.052	3.580*	0.046*
	CMD	15.714*	0.002*	2.195	0.136
**Shoulder ab/adduction**	RoM	13.268*	0.004*	4.424*	0.024*
	SD	16.658*	0.002*	0.921	0.413
	CMD	1.048	0.328	9.782*	0.001*
**Elbow flexion/extension**	RoM	0.092	0.767	3.067	0.068
	SD	3.568	0.085	0.117	0.890
	CMD	8.341*	0.015*	1.813	0.187
**Trunk transverse rotation**	RoM	23.133*	0.001*	0.359	0.702
	SD	14.062*	0.003*	2.470	0.108
	CMD	1.058	0.325	0.386	0.684
**Trunk lateral flexion**	RoM	21.476*	0.001*	3.653*	0.044*
	SD	35.563*	<0.001*	1.393	0.271
	CMD	0.901	0.362	2.737	0.087
**Trunk forward flexion**	RoM	40.988*	<0.001*	4.263*	0.028*
	SD	4.030	0.700	2.670	0.092
	CMD	7.040*	0.012*	1.204	0.313

Significant effects (p ≤ 0.05) are denoted by an asterisk.

## Discussion

Controlled goal-oriented tasks that reflected ADLs were executed by prosthesis users and able-bodied controls to determine if: 1) the loss of active distal DoFs associated with a transradial prosthesis encouraged alterations in kinematic RoM, 2) transradial prosthesis users demonstrated increased kinematic variability, and 3) prosthesis experience was associated with increased kinematic repeatability.

### Degrees-of-freedom range-of-motion

Prosthesis users demonstrated greater shoulder abduction and trunk RoM than the able-bodied controls when observed across tasks (Figure 3). Generally, the kinematic profiles of prosthesis users were distinct from those of able-bodied individuals, and Figure 2 provides an example of the unique management of upper body DoFs for prosthesis users. The use of available trunk DoFs to compensate for loss of active distal arm DoFs of transradial prosthesis users has been reported previously [5, 6] and these results agree with the literature. Given that the ability to manipulate end point position in the sagittal plane is not affected by use of a transradial prosthesis, the absence of significant differences in shoulder and elbow flexion/extension RoM seems reasonable. Similar to individuals post-stroke and with frozen shoulder, transradial prosthesis users appear to be using the trunk as part of the kinematic chain to manipulate endpoint position [4–8]. The combination of increased shoulder abduction and trunk motion facilitates execution of the required tasks for prosthesis users, as these DoFs working in synergy would theoretically compensate for absence of active supination/pronation of the forearm. Although there is some evidence to suggest that trunk restraint training reduces trunk compensatory motions and elicits modifications in arm kinematics to facilitate task execution in individuals post-stroke [42–44], the complete loss of active distal DoFs in transradial prosthesis users likely requires consistent recruitment of the trunk DoFs. Given that transradial prosthesis users possess an internal model of the affected limb to drive motor patterns that are comparable to normal physiologic function [16–19], this further suggests that trunk and shoulder compensatory motions are essential voluntary behavior to facilitate task execution.

Overall, these results seem to support the idea that it may be advantageous to encourage compensatory movements in a manner that is optimally ergonomic to facilitate task execution and minimize injury, and this may have implications for prosthetic training. The recruitment of trunk and shoulder motion demonstrates the flexibility of the upper body motor system and the adaptability of transradial prosthesis users when executing ADLs. Training that encourages successful management of the DoFs of the upper body could counter the functional limitations of currently available transradial prostheses, and although compensatory movements may impose unfavorable metabolic costs, the functional improvements may ultimately enhance user perception of device utility [25–27]. However, as there is indication that sustained trunk forward flexion compared to upright posture [45] and iso-directional coupled arm movement compared to anti-directional coupling [46] during standing increases metabolic cost, future research should investigate the impact of upper body compensatory motions and posture on metabolic cost during upper body goal-oriented task execution. Furthermore, with advances in upper limb prosthetic control techniques for active wrist rotation [47], the contribution of this voluntary DoF to upper body compensatory movements warrants investigation.

### Kinematic variability

Apart from trunk forward flexion, the DoFs that displayed increased RoM in prosthesis users also demonstrated a significant increase in within-subject SD compared to able-bodied controls. These results suggest that these compensatory dynamics appear to be associated with an increase in average variability of movement, such that prosthesis users generally do not converge on a defined motor synergy to the degree observed in able-bodied individuals. The DoFs that did not demonstrate a significant increase in SD for prosthesis users, which would otherwise indicate greater variability, displayed a significant increase in CMD for able-bodied controls. This result suggests greater kinematic repeatability of controls compared to prosthesis users for those DoFs, and also the complementary information provided by the SD and CMD techniques. The increased shoulder and trunk variability of prosthesis users did not preclude execution of tasks 3, 4, and 5, and this further demonstrates the utility of the motor flexibility associated with the upper body kinematic chain to manipulate end point position. Consequently, this variability may be seen as advantageous when operating a transradial prosthesis with reduced active distal DoFs and reflective of healthy motor performance [24, 48], where the various DoFs are able to compensate for each other to ensure repeated task execution. However, this motor flexibility is not without limitation, as the majority of participants were unable to rotate a page 180 degrees as required by page turning in a book (task 2).

Increased variability may also be viewed as diminished movement quality when compared to able-bodied individuals, as real-time adaptation during execution was not necessary since there were no external factors such as perturbation. Although the tasks executed in this study did not require specific joint/segment configurations, able-bodied individuals were able to maintain relatively low levels of within-subject absolute variability (Figure 5) and while only significant for some DoFs, prosthesis user variability averages were consistently greater across all tasks and DoFs. Increased movement variability may consequently be identified by prosthesis users as inconsistent or unreliable device response, and hence increase end-user frustration and diminish the perceived utility of the device as has been demonstrated during human interaction with computers via a controller [49, 50]. Frustration may be a realistic outcome if prosthesis users perceive kinematic variability as an obstacle to consistent goal attainment, and anecdotally, such a response was observed in this study when some users were unable to reposition the knife in their prosthesis to replicate execution of task 1.

Overall kinematic repeatability shared a direct relationship with prosthesis experience. As only prosthesis users were tested in this study and only during one testing session, the causal relationship between experience and variability is unknown, i.e., whether kinematic variability decreases over time with device experience, or low levels of variability encourage continued device use. However, motor adaptation and learning through practice and experience is essential for upper extremity neurorehabilitation to minimize variable outcomes and maximize likelihood of successful task execution [23]. Similarly, there is some evidence to suggest that an inverse relationship exists between athletic skill and movement variability for those dynamics responsible for performance [51–53]. Consequently, it may be reasonable to postulate that kinematic variability of goal-oriented tasks would decrease with training and experience, such that those individuals with greater experience would possess an enhanced ability to integrate various forms of sensory feedback to execute tasks with near normal reliability [20, 54]. In fact, the linear trend in experience and kinematic repeatability was such that as experience increased, repeatability approached values equivalent to that of able-bodied controls. These results further encourage investigations into the relationships between kinematic variability, device experience, training modalities, and user satisfaction.

A limitation of this study is that due to the small sample size, which reflects the practicalities of *in-vivo* prosthetic upper limb research given a small overall population size, prosthetic designs varied between participants. Although there is limited evidence to suggest that there is no difference in functional performance between single DoF and multi-articulated prosthetic hands [55], the use of different devices may have introduced a confounding factor to kinematic performance. However, the mixed model analysis helped account for within-subject variability across tasks and thereby lends confidence that the statistical results reflect true differences between groups. Additionally, only a selection of tasks were executed by participants in this study and, although they reflect ADLs, generalization of the results should be made with caution based on this limitation. Finally, the assessments of kinematic variability to produce single statistical measures were based on time-normalized kinematic profiles that are unable to identify the individual contributions of phase and magnitude differences. Other, more sophisticated, methods to quantify these aspects of variability, such as dynamic time-warping [56], may offer additional insight into this outcome measure.

## Conclusions

Transradial prosthesis users utilize shoulder abduction and trunk movement as compensatory motions to execute goal-oriented tasks, and the majority of these motions are accompanied by increased kinematic variability when compared to able-bodied controls. The average repeatability of upper body kinematics was positively associated with prosthesis experience. As these dynamics may be necessary to compensate for the absence of active distal DoFs in the prosthetic arm, transradial prosthesis users may benefit from dedicated training that: 1) encourages optimization of these dynamics to facilitate execution of ADLs, and 2) fosters adaptable but reliable motor strategies. Importantly, results from this study further emphasize the utility of motion capture for investigating movement quality of upper limb prosthesis users, which can complement information on functional clinical outcomes when assessing overall performance. As new upper limb prosthetic designs with increased active DoFs become commercially available, more sophisticated outcome metrics that consider functional clinical outcomes and movement quality may be necessary to effectively assess the contribution of these design features to user performance.

## Authors’ original submitted files for images

Below are the links to the authors’ original submitted files for images. Authors’ original file for figure 1 Authors’ original file for figure 2 Authors’ original file for figure 3 Authors’ original file for figure 4 Authors’ original file for figure 5

## Abbreviations

DoF: Degree-of-freedom
CNS: Central nervous system
ADL: Activity of daily living
RoM: Range-of-motion
SHAP: Southampton Hand Assessment Procedure
SD: Standard deviation
CMD: Adjusted coefficient of multiple determination
LMM: Linear mixed model.
