# Supplementary figures and images for: Comparison of range-of-motion and variability in upper body movements between transradial prosthesis users and able-bodied controls when executing goal-oriented tasks
Source: J Neuroeng Rehabil. 2014 Sep 6;11:132. doi: 10.1186/1743-0003-11-132 (PMC4164738; doi:10.1186/1743-0003-11-132)

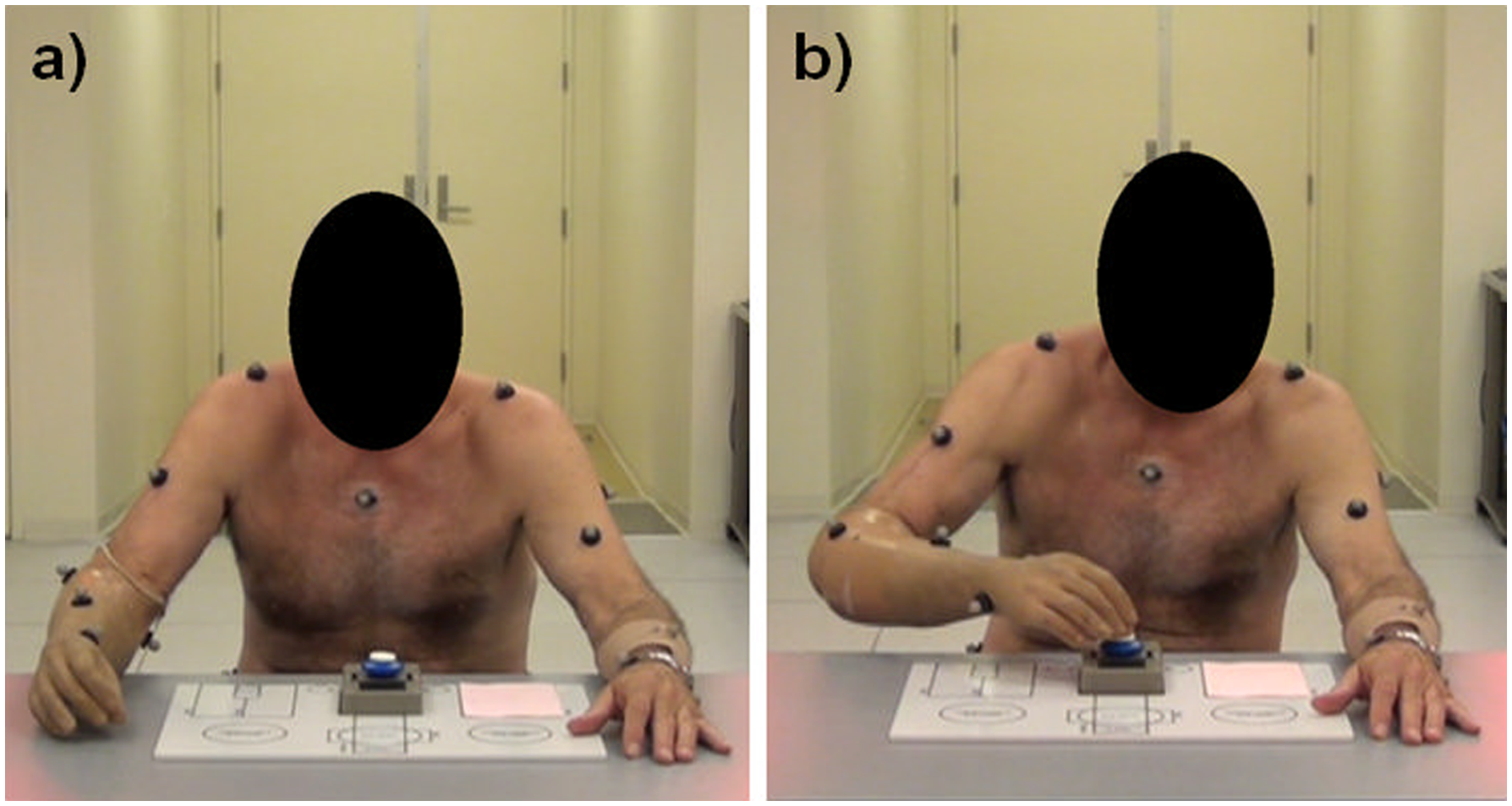

Supplement: Supplementary file 1 — Authors’ original file for figure 1 [file 12984_2014_654_MOESM1_ESM.tif]

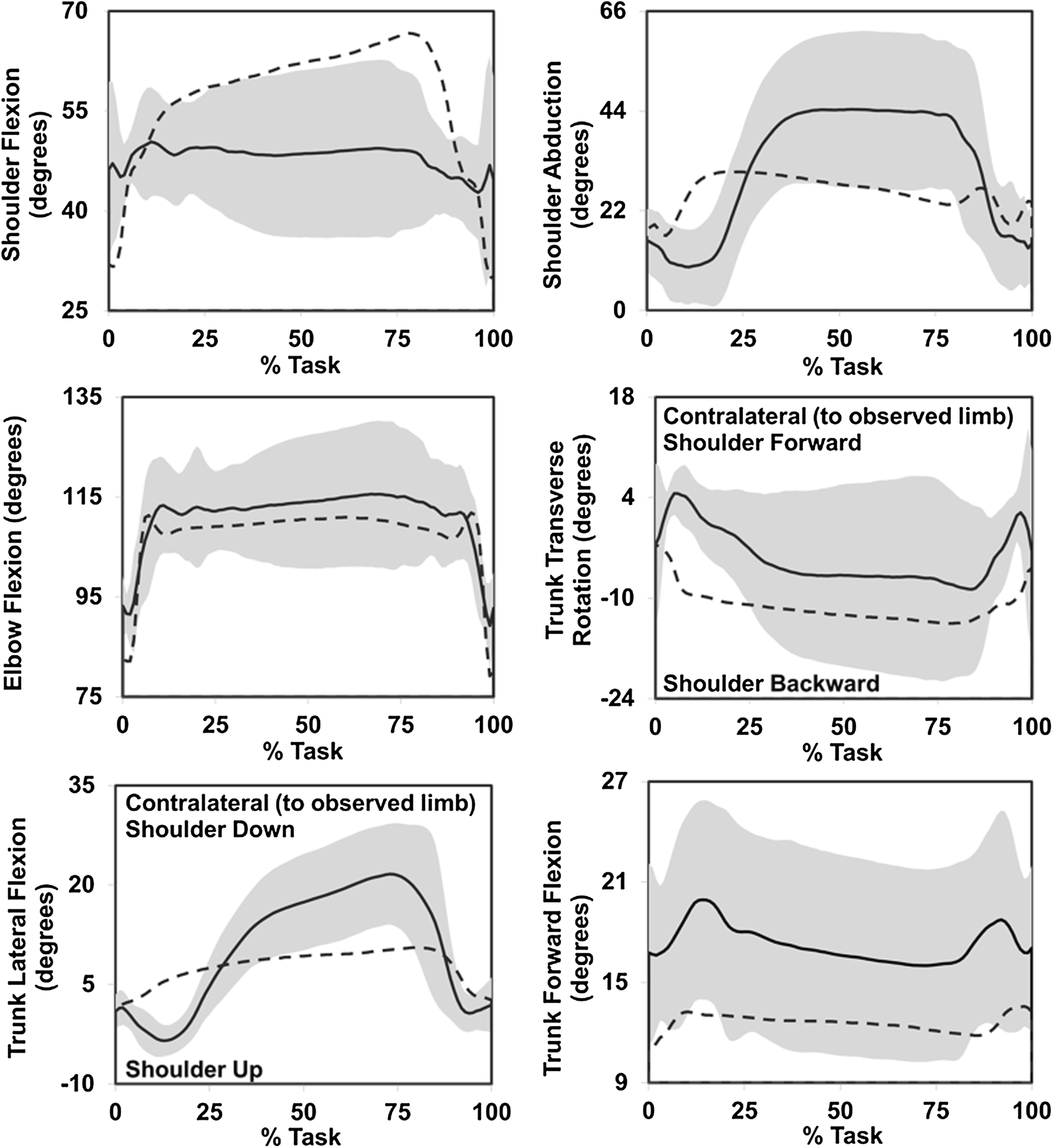

Supplement: Supplementary file 2 — Authors’ original file for figure 2 [file 12984_2014_654_MOESM2_ESM.tiff]

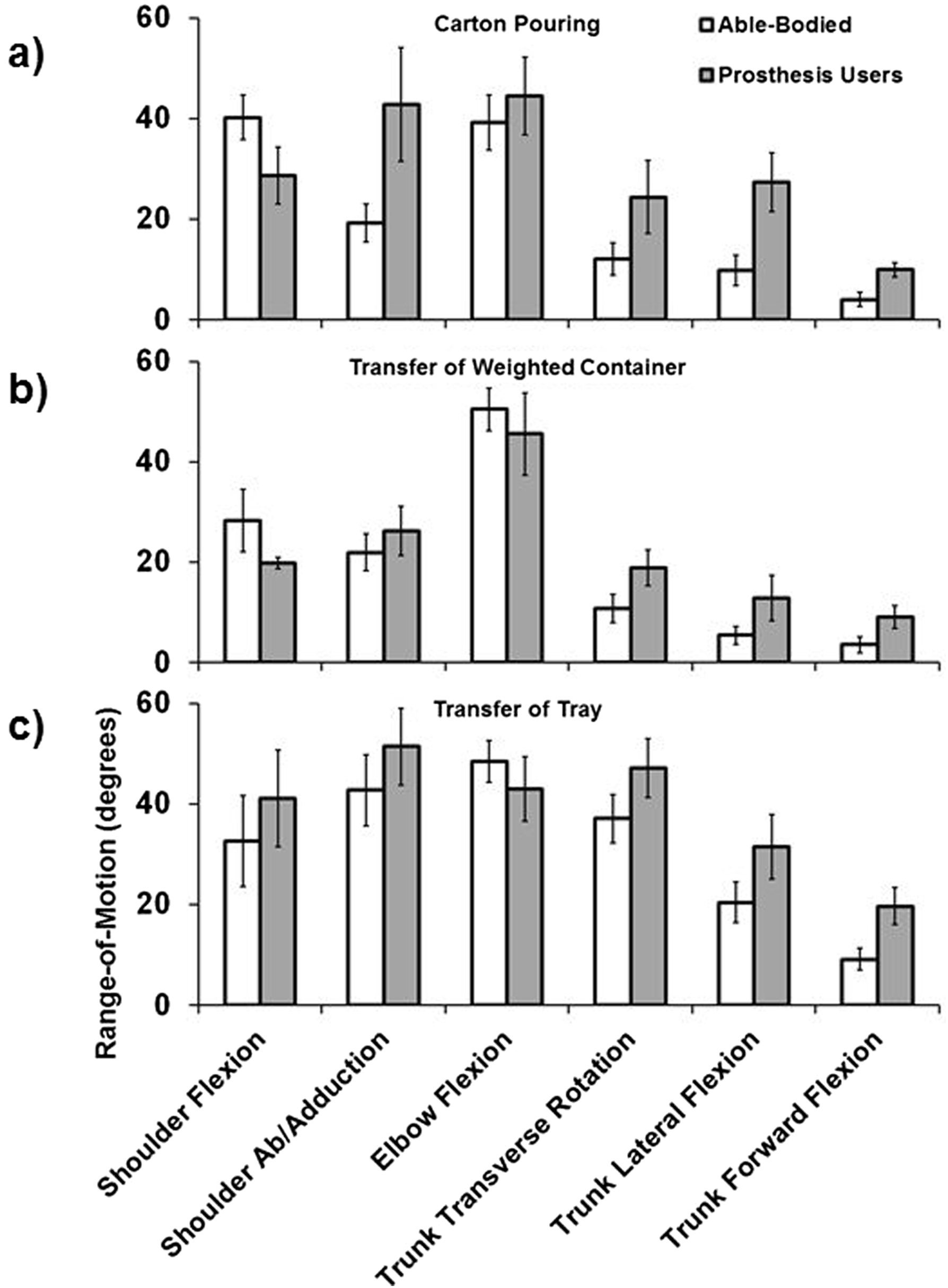

Supplement: Supplementary file 3 — Authors’ original file for figure 3 [file 12984_2014_654_MOESM3_ESM.tif]

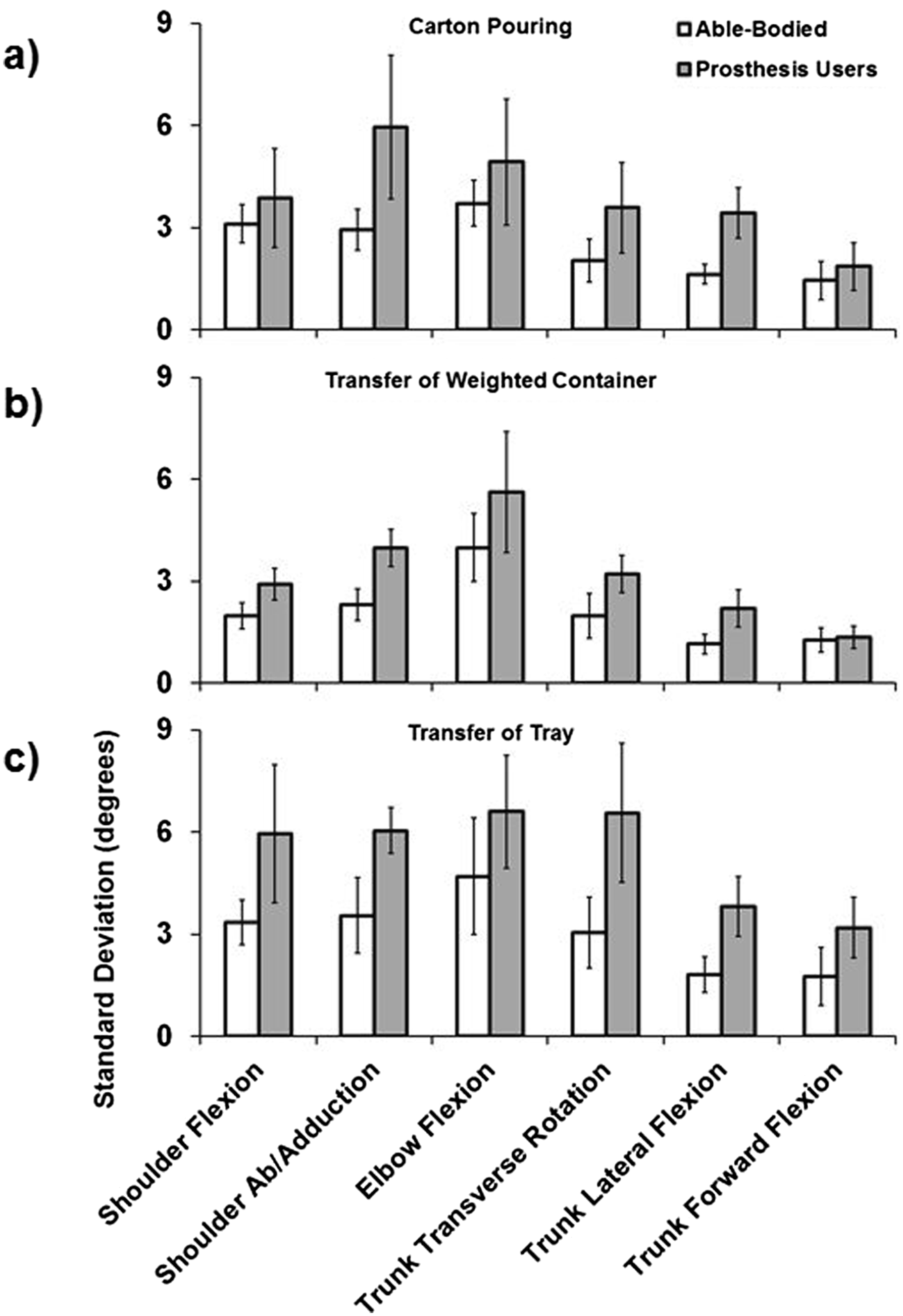

Supplement: Supplementary file 4 — Authors’ original file for figure 4 [file 12984_2014_654_MOESM4_ESM.tif]

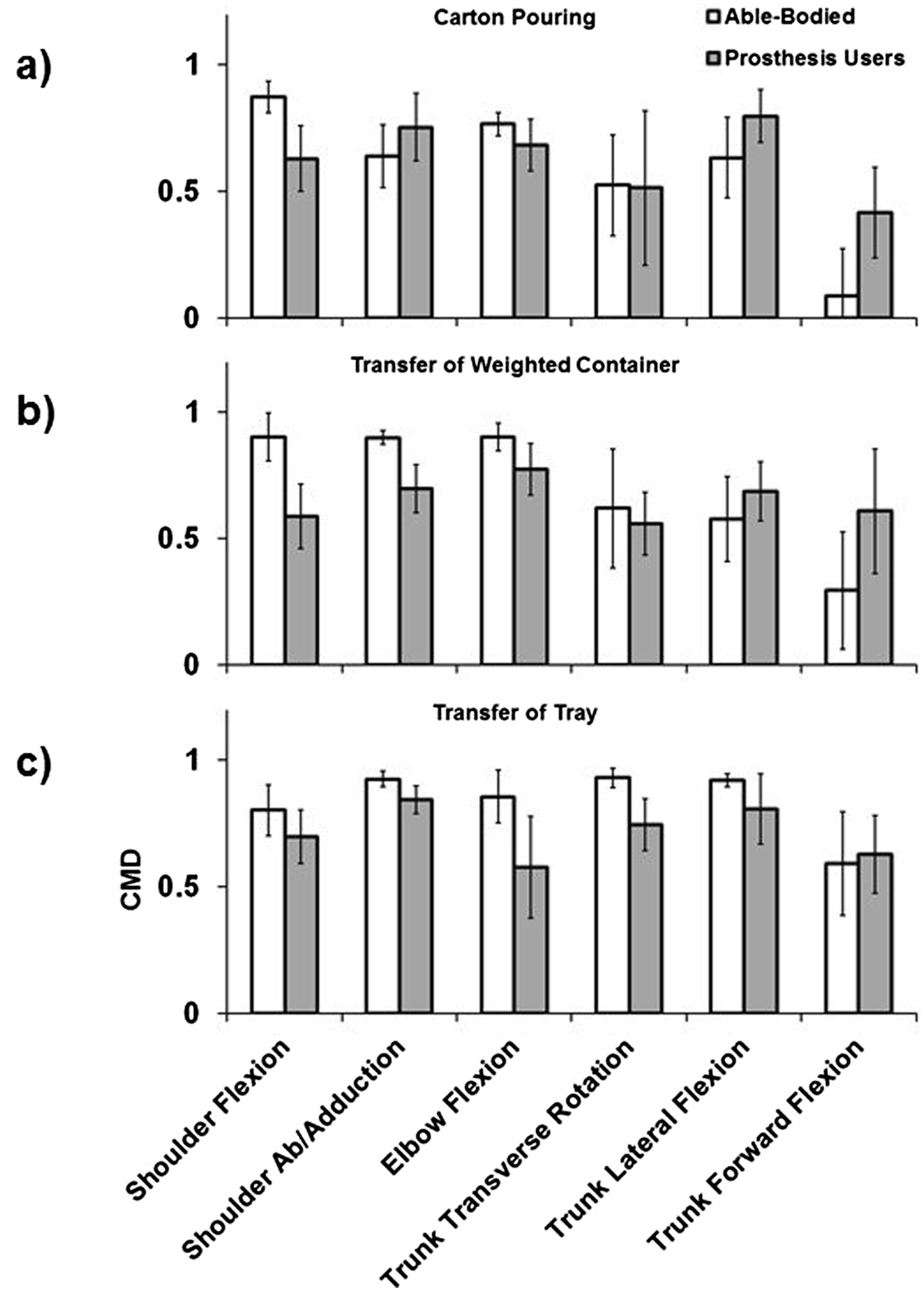

Supplement: Supplementary file 5 — Authors’ original file for figure 5 [file 12984_2014_654_MOESM5_ESM.tif]
